# Angio-embolization of a renal pseudoaneurysm complicating a percutaneous renal biopsy: a case report

**DOI:** 10.11604/pamj.2015.22.278.7976

**Published:** 2015-11-23

**Authors:** Hicham Rafik, Mounia Azizi, Driss El Kabbaj, Mohammed Benyahia

**Affiliations:** 1 Department of Nephrology, Mohammed V Military Hospital, Faculty of Medicine and Pharmacy University Mohammed V-Souissi, Rabat, Morocco

**Keywords:** Angioembolization, renal pseudoaneurysm, kidney

## Abstract

We report the treatment of a bleeding renal pseudoaneurysm by angio-embolization. A 21 years old woman developed macroscopic haematuria following renal biopsy. Renal angio-scan showed a 1.4 cm renal pseudoaneurysm in the left kidney. The presence of pseudoaneurysm was confirmed by selective renal angiography. Successful embolization was performed using gelatine sponge particles.

## Introduction

The percutaneous renal biopsy remains the standard method of acquiring renal tissue. It is an essential tool in the diagnosis, and management of renal disease in native and transplanted kidneys. Complications, although rare, may occur and the majority of these are related to bleeding [1]. Pseudoaneurysms are a rare complication of renal biopsy. These lesions are unstable and their ruptured can lead to life threatening hemorrhage. The treatment of choice is endovascular selective angio-embolization, although surgery might be indicated when hemostasis and repair of the arterial wall defect are required [2]. We present a case of massive haematuria due to renal pseudoaneurysm developing after renal biopsy that was managed with angiographic embolization.

## Patient and observation

**Figure 1:**
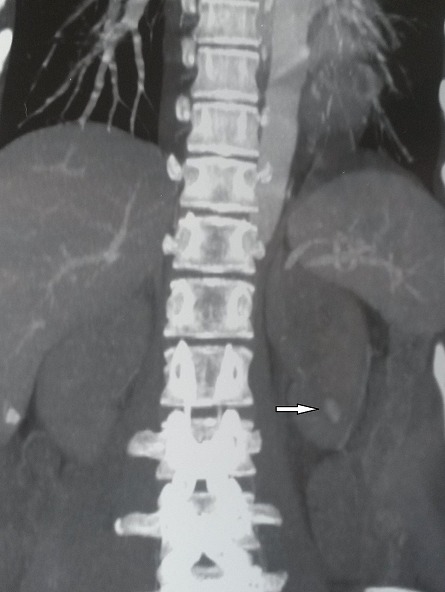
Renal angio-scan shows a 1.4 cm renal pseudoaneurysm in the left kidney

**Figure 2:**
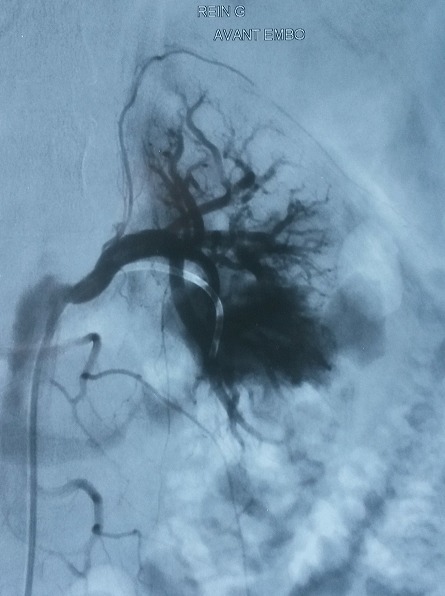
Renal angiography: extravasation of CO_2_ in the pseudoaneurysm situated in the lower pole

**Figure 3:**
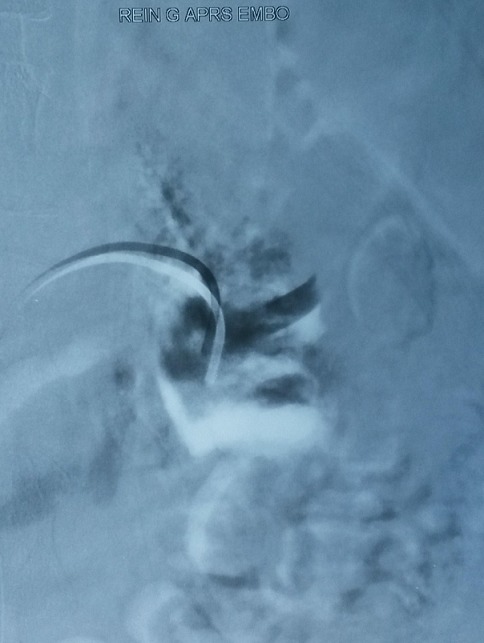
Embolization of pseudoaneurysm selectively with gelatine sponge particles

A 21-years-woman was referred to the division of nephrology for a nephrotic syndrome. She received a percutaneous ultrasound -guided renal biopsy to determine the cause of nephrotic syndrome. About 4 hours after the biopsy, the patient developed massive gross haematuria and urinary clot retention.On examination, her blood pressure was 90/60 mmHg and her pulse was 110heats/minute. Hemoglobin concentration had dropped from 10g/dl to 6g/dl. There were no bruits or palpable masse.The patient regained hemodynamic stability after a 4 U blood transfusion. An ultrasound showed a blood flow containing round structure compatible with a pseudoaneurysm next to the biopsy site at the lower pole of the biopsied kidney. Renal angio-scan revealed a 1,4 cm-sized renal pseudoaneurysm (Figure 1). Renal angiography confirmed the presence of a pseudoaneurysm with extravasation of Co_2_ from the lower left interlobar artery (Figure 2). Successful selective embolization of the pseudoaneurysm was performed using gelatine sponge particles (Figure 3). There was no further haematuria. Nevertheless, left lumbago was appeared after embolization, and it is effectively treated with analgesic. Three days after the procedure, follow up Doppler ultrasonography showed no blood flow into the aneurysmal sac.

## Discussion

Renal biopsy is a routine examination method in nephrology. Its development was enabled by an increased usage of ultrasonography and by introducing automated biopsy sets. The number of all complications accompanying kidney biopsy falls within the range of 5 to 10%. Among the most frequent complications we may find microscopic haematuria (25%) and perirenal haematoma (42%) [3].

Renal pseudoaneurysm has been reported to occur in various clinical scenarios, including after renal trauma, surgery and percutaneous procedures, as well as inflammatory and neoplastic processes within the kidney [2]. Reports of patients developing pseudoaneurysm after percutaneous renal biopsy within any kidney-related surgery or trauma are very rare. Most of the reported cases occurred in renal transplant recipient after renal allograft biopsy or in those with a surgical history such as partial nephrectomy [4–6]. This patient had no history of trauma, surgery, neoplasm, or autoimmune disease.

From a series of 72 consecutives percutaneous allograft biopsies studied with duplex ultrasonography four patients (5.6%) were found to have pseudoaneurysm. All pseudoaneurysms closed spontaneously [7]. Tondel published the largest report of renal biopsy complication: 9288 biopsies from the Norwegian kidney biopsy registry, 0.9% of the patients needed blood, 0.2% required an invasive procedure (surgery a angio intervention), and 1.9% had a macroscopic hematuria [8]. Patients may complain of flank pain or gross hematuria and may even present with anemia or shock.[2] In the current case, the patient complained of gross total hematuria and retention of urine.

A diagnosis of the renal pseudoaneurysms can be made based on non-invasive methods and these include an ultrasonography, a computed tomography and a magnetic resonance imaging. An angiogaphy of the renal artery is an invasive method but it is useful to provide obscure anatomical data and to treat the corresponding cases [9]. In this case, renal angio-scan was performed for evaluation of gross haematuria. Eventually, pseudoaneurysm was identified and then treated using renal angiography.

Treatment modalities for the renal pseudoaneurysm include observation, non-surgical methods such as angiographic arterial embolization and surgical methods such as nephrectomy or partial nephrectomy [9]. Selective angioembolization is first-line therapy for renal pseudoaneurysm.The procedure is safe and effective and minimizes the territory of infarction [2, 5, 6]. To avoid nephrectomy at our patient, we attempted embolization by setting up gelatine sponge particles, successfully.

## Conclusion

According to the results of the current case, the selective embolization of the renal artery branch is an effective treatment for renal pseudoaneurysm.
